# Bodily awareness predicts functional improvement and injury risk in elite long-distance runners: a prospective study

**DOI:** 10.3389/fpsyg.2025.1718718

**Published:** 2025-12-17

**Authors:** Koji Murofushi, Sho Mitomo, Yosuke Yamada, Kenji Hirohata, Hidetaka Furuya, Hiroki Katagiri, Hiroshi Akuzawa, Susumu Hara, Masakazu Ito, Koji Kaneoka

**Affiliations:** 1 Sports Science Center, Institute of Science Tokyo, Tokyo, Japan; 2 Department of Orthopedic and Spinal Surgery, Graduate School of Medical and Dental Sciences, Institute of Science Tokyo, Tokyo, Japan; 3 Sports and Health Sciences, Graduate School of Biomedical Engineering, Tohoku University, Sendai, Japan; 4 Clinical Center for Sports Medicine and Sports Dentistry, Institute of Science Tokyo, Tokyo, Japan; 5 Department of Rehabilitation, Sonoda Third Hospital, Tokyo, Japan; 6 Tokyo Medical Institute Tokyo Spine Center, Tokyo, Japan; 7 Department of Orthopedics, Dokkyo Medical University Saitama Medical Center, Saitama, Japan; 8 Athlete Support Research Center, Niigata University of Health and Welfare, Niigata, Japan; 9 School of Global Studies and Collaboration, Aoyama Gakuin University, Tokyo, Japan; 10 Faculty of Sport Science, Waseda University, Tokyo, Japan

**Keywords:** bodily awareness, corrective exercise, functional screening, injury prevention, interoception, long-distance runners, running-related injuries

## Abstract

Running-related injuries (RRIs) are prevalent among long-distance athletes. Although physical conditioning remains central to preventing RRIs, bodily awareness may play an equally important role in self-regulation. The KOJI AWARENESS (KA) test was designed to enhance awareness by enabling athletes to evaluate their functional status. This study investigated whether bodily awareness, assessed by the KA test, is associated with functional improvement and reduced RRI incidence in male collegiate long-distance runners. A total of 41 male collegiate runners were recruited (mean age: 20 ± 1 years, height: 171.6 ± 5.9cm, weight: 56.6 ± 4.1kg), and 35 completed the protocol. All participants underwent pre- and post-season KA testing and performed individualized corrective exercises based on their results. Awareness of functional decline was measured at only post-season by questionnaire with a five-point Likert scale, with athletes selecting “Very aware” classified into the Excellent Awareness Group. KA scores, score changes, and RRI incidence were compared between groups. Eight runners (22.9%) demonstrated excellent awareness; this group had significantly higher post-season KA scores and greater absolute and relative improvements than the Not-Excellent Awareness Group (*p* < 0.05). RRI incidence was 0% in the Excellent Awareness Group and 14.8% in the other group, though the difference was not statistically significant. Overall RRI incidence was 11.4%. These findings suggest that cultivating bodily awareness strengthens the impact of corrective interventions. Awareness may act as a bridge between movement, perception, and resilience to injury in athletic performance.

## 1 Introduction

Running is a widely practiced physical activity that supports overall health and well-being (Lee et al., 2017; Nielsen et al., 2024). Systematic review indicates that the prevalence of running-related injuries (RRIs) among long-distance runners ranges from 19.4 to 79.3% (van Gent et al., 2007). One review reported that 44.6 ± 18.4% of runners sustain musculoskeletal injuries, with Achilles' tendinopathy (10.3%), medial tibial stress syndrome (9.4%), and patellofemoral pain syndrome (6.3%) being the most common (Kakouris et al., 2021). A separate study of competitive runners further noted that 62.6% of them had experienced RRIs (Fredette et al., 2022). Beyond training-related variables, physical impairments such as hip and core weakness or pes planus are closely linked to RRI incidence (Mucha et al., 2017; De Blaiser et al., 2019; Rabin et al., 2014). These findings highlight the importance of assessing and understanding individual physical function to prevent injuries in competitive runners.

Enhancing athletes' awareness of their movement patterns and physical function reduces compensatory movement, lowers re-injury rates, and fosters self-directed behavioral change (Wang et al., 2023; Montero, 2015). Kesilmiş et al. demonstrated that athletes with greater physical awareness performed better at detecting performance limitations, which correlated with motivational goal orientations (Kesilmiş and Yildiz, 2018). Research on psychological skill training, including imagery, also suggests a close connection between body awareness and athletic performance (Biscardi et al., 2024). Awareness, therefore, is not merely cognitive but a prerequisite for motor control and safe, efficient movement (Wolpert and Ghahramani, 2000; Matsumiya, 2021).

The KOJI AWARENESS (KA) screening test was developed to help athletes identify functional impairments through perceptual engagement (Murofushi et al., 2022a,b, 2024a,c; Takasaki and Kanayasu, 2024). This tool allows athletes to take an active role in evaluating their physical condition. Murofushi et al. found that runners with lower pre-season KA scores had significantly higher in-season RRI rates, and that a KA score threshold of ≤ 46.5 corresponded to a 2.65-fold greater injury risk (Murofushi et al., 2024c). These results suggest that KA testing may support both injury-risk screening and the promotion of preventive behaviors through enhanced self-awareness. However, intervention outcomes often vary depending on athletes' baseline awareness. Practitioners frequently note that athletes with greater awareness achieve better results, yet the association between KA scores and intervention efficacy remains unclear.

Awareness of one's body is a central concept in both Western phenomenology and Eastern traditions (Merleau-Ponty, 1962; Heidegger, 1962; Wallman-Jones et al., 2021). Rather than treating the body as an object, these traditions view bodily awareness as the foundation of perception and action. This aligns with the principles underlying the Koji Awareness (KA) test, where the goal is to enhance athletic performance by improving bodily self-awareness and control. Recent research also supports that such awareness regulates effort and movement in physical activity, highlighting its empirical and clinical relevance in sports contexts. This theoretical grounding suggests that awareness, as conceptualized in both traditions, may play a pivotal role in functional assessments such as the KA test.

This study aimed to examine the relationship between physical awareness—measured by the KA test—and the effectiveness of exercise interventions in reducing RRIs and improving in-season KA scores among long-distance runners. We hypothesized that players who gained awareness of their physical capabilities through pre-season KA score measurements showed greater improvement in their KA scores after the season and had a lower incidence of RRIs during the season.

## 2 Materials and methods

### 2.1 Participants

This study included 41 male collegiate long-distance runners (mean age: 20 ± 1 years, height: 172.6 ± 5.9cm, weight: 56.6 kg) from a university Ekiden team competing in the All-Japan University Ekiden Championship (Hakone Ekiden) in the 2023 and 2024 seasons. The Hakone Ekiden is a relay race where 10 runners cover 217.1 km. This team won the Hakone Ekiden in the 2023 and 2024 with the time of 10 h, 41 min, and 25 s in 2023 season and 10 h, 41 min, and 25 s in 2024 season. Exclusion criteria were as follows: (i) severe psychiatric, neurological, or cardiovascular disorders; (ii) orthopedic conditions; and (iii) acute infectious diseases. Prior to testing, all participants completed a pre-assessment questionnaire addressing physical characteristics (height, weight, sex, age), medical and athletic history, participation level, and daily activity habits. Written informed consent was obtained from all participants. Testing was discontinued immediately if participants experienced pain.

This study followed the ethical principles outlined in the Declaration of Helsinki (52nd WMA General Assembly, Edinburgh, Scotland, October 2000) for medical research involving human participants. The Research Ethics Committee of the Institute of Science Tokyo approved the protocol (Approval ID: M2019-168).

### 2.2 Procedures

Participant characteristics, including age, height, weight, and body mass index (BMI), were recorded. Six months before the Hakone Ekiden season, participants completed the KA test and a self-awareness questionnaire. Based on KA results, corrective exercises were prescribed and performed throughout the competitive season. The KA test was repeated after the Hakone Ekiden. Participants were also monitored for RRIs over 6 months, and any missed training sessions due to injury or insufficient conditioning were documented.

### 2.3 Qualitative evaluation of awareness and group classification

Participants were surveyed at post-season regarding their awareness of functional decline using the prompt: “To what extent did the KA test help you become aware of problematic or weak areas in your body?”

Responses were collected on a five-point Likert scale: “Very aware,” “Somewhat aware,” “No change,” “Not very aware,” and “Not aware at all.” Participants who responded “Very aware” were assigned to the Excellent Awareness Group, while all others were classified as the Not-Excellent Awareness Group.

Post-season assessment of bodily awareness was conducted to capture cumulative effects of corrective exercises and seasonal training on interoceptive perception (Mehling, 2020). This timing offers an ecologically valid view of athletes' functional state and helps determine whether adaptations persist and reflect integrated physiological changes (Zeng et al., 2025; Lischke et al., 2021).

### 2.4 KA test

The KA test comprises 11 components that assess mobility, stability, strength, and balance (Murofushi et al., 2022a, 2024a,b,c; Takasaki and Kanayasu, 2024). It provides a functional evaluation of multiple body regions without the use of specialized or invasive equipment. Each component is organized by anatomical segments to facilitate the identification of dysfunction, with clearly defined scoring criteria. The maximum total score is 50 points. Murofushi et al. (2022b) described the details of the procedure.

The KA test was administered at two time points: 6 months before the Hakone Ekiden (pre-season) and immediately after the competition (post-season). Assessments were conducted by each athlete to raise awareness of their body under the supervision of an American College of Sports Medicine–certified exercise physiologist. Both total and segmental scores were calculated. Following prior studies (Murofushi et al., 2024a), segmental scores were computed for the neck-scapula-upper extremity complex (NSU), trunk, and lower extremity (LE; Table 1).

**Table 1:** Breakdown of each test item and score in the KA screening test, distribution of each segment, and corrective exercises for the corresponding functional deficiency.

**Section**	**Test**	**Total score**	**Segment**	**Corrective exercises for the corresponding functional deficiency**
1	Neck mobility	6	NSU	Archer's rotation Python squeeze
2	Shoulder mobility	2	NSU	Wall reverse push
3	Shoulder blade (scapular mobility)	2	NSU	Wall angel slider
4	Thoracic spine mobility	6	Trunk	Flamenco thoracic spine rotation
5	Upper extremity stability and strength	4	NSU, Trunk	Weight shift wall push
6	Hip mobility	8	LE	Weight shift squat Side sitting to lift up
7	Hip and spine mobility	6	Trunk, LE	Straight leg lowering Single-leg squat with ankle hold
8	Upper and lower extremity mobility and stability	2	Trunk, LE	Single-leg squat with ankle hold
9	Mid-section stability strength	4	Trunk	Straight leg lowering 45
10	Lower extremity strength	8	LE	Weight shift squat Single-leg squat with ankle hold
11	Ankle mobility	2	LE	Koji wall push
Total score		50		

KA, KOJI AWARENESS; LE, lower extremity; NSU, neck-scapula-upper extremity-complex.

The NSU segment assessed cervical spine, scapular, and upper extremity mobility and strength. The trunk segment evaluated thoracic spine mobility, trunk muscle strength and stability, and mobility and stability across both upper and lower extremities. The LE segment comprised measures of hip and spinal mobility, hip and ankle mobility, LE strength, and overall stability. Each segment score was defined by the sum of its relevant subcomponents.

Further, the NSU segment included neck mobility, shoulder mobility, scapular mobility, and upper extremity stability and strength, with a maximum score of 14 points. The trunk segment comprised thoracic spine mobility, upper extremity stability and strength, hip and spinal mobility, upper and lower extremity mobility and stability, and mid-section stability and strength, with a maximum score of 22 points. The LE segment included hip mobility, hip and spinal mobility, upper and lower extremity mobility and stability, LE strength, and ankle mobility, with a maximum score of 26 points.

Upper extremity stability and strength, hip and spinal mobility, and upper and lower extremity stability were considered relevant to both adjacent segments (e.g., trunk–limb coordination) and were therefore included in multiple segment scores as appropriate.

From pre-season and post-season KA test results, both absolute and percentage changes were calculated for total and segmental scores. The KA test has a demonstrated high reproducibility, with an intraclass correlation coefficient (1.1) of 0.876 (95% confidence interval [CI]: 0.434–0.981; Murofushi et al., 2024a).

### 2.5 Intervention through supervised corrective exercise

KA corrective exercises are designed to target the specific body parts exhibiting corresponding functional impairments (Murofushi et al., 2024b, 2025; see Appendix). Exercises were individually prescribed for all participants based on their KA test performance, for which participants lost points measured by KA self-screening. Corresponding exercises for each functional deficiency in each body part were summarized in Table 1. The exercises were implemented at the university athletic field three times per week under the supervision of the head and assistant coaches, and the certified exercise physiologist. The exercises were incorporated into the team's warm-up routine and targeted identified problem areas prior to regular training sessions. Integration into the structured warm-up protocol facilitated sustained motivation and ensured consistent adherence among all participants.

### 2.6 Definition and classification of RRIs

During the competitive season, RRIs were recorded for each participant and minor discomforts or subclinical symptoms that did not lead to a formal diagnosis were also monitored. For the purpose of classification, an RRI was defined in accordance with previous studies (van Gent et al., 2007; Kakouris et al., 2021; Murofushi et al., 2024c; Takasaki and Kanayasu, 2024) as a sports-related injury or trauma to the trunk or LE triggered by running, thereby resulting in absence from training or competition for >3 weeks.

### 2.7 Injury surveillance

Following KA testing, RRI incidence was prospectively monitored over the 6-month competitive season. For each injury, the specific diagnosis and number of days absent from training and competition were recorded. Absence duration was defined as the number of days from the first missed session until full return to training and competition. All injuries were documented by a certified athletic trainer assigned exclusively to the team.

### 2.8 Statistical analysis

Normality of variable distributions was assessed using histograms and the Shapiro–Wilk test. Descriptive statistics were reported as mean ± standard deviation for normally distributed variables, and as median with interquartile range for non-normally distributed variables. Between-group differences based on awareness level were examined using independent samples t-tests or Mann–Whitney U tests for participant characteristics, pre-season and post-season KA scores, and their absolute and relative changes. The Fisher's exact test evaluated differences in RRI incidence between awareness groups and the risk ratio was calculated. The effect size (ES, Cohen's *d* or *r* value) was calculated in the *post hoc* test. All analyses were performed with SPSS Statistics version 27 (IBM Corp., Armonk, NY, USA), with the significance level set at 5%.

## 3 Results

**Figure 1:**
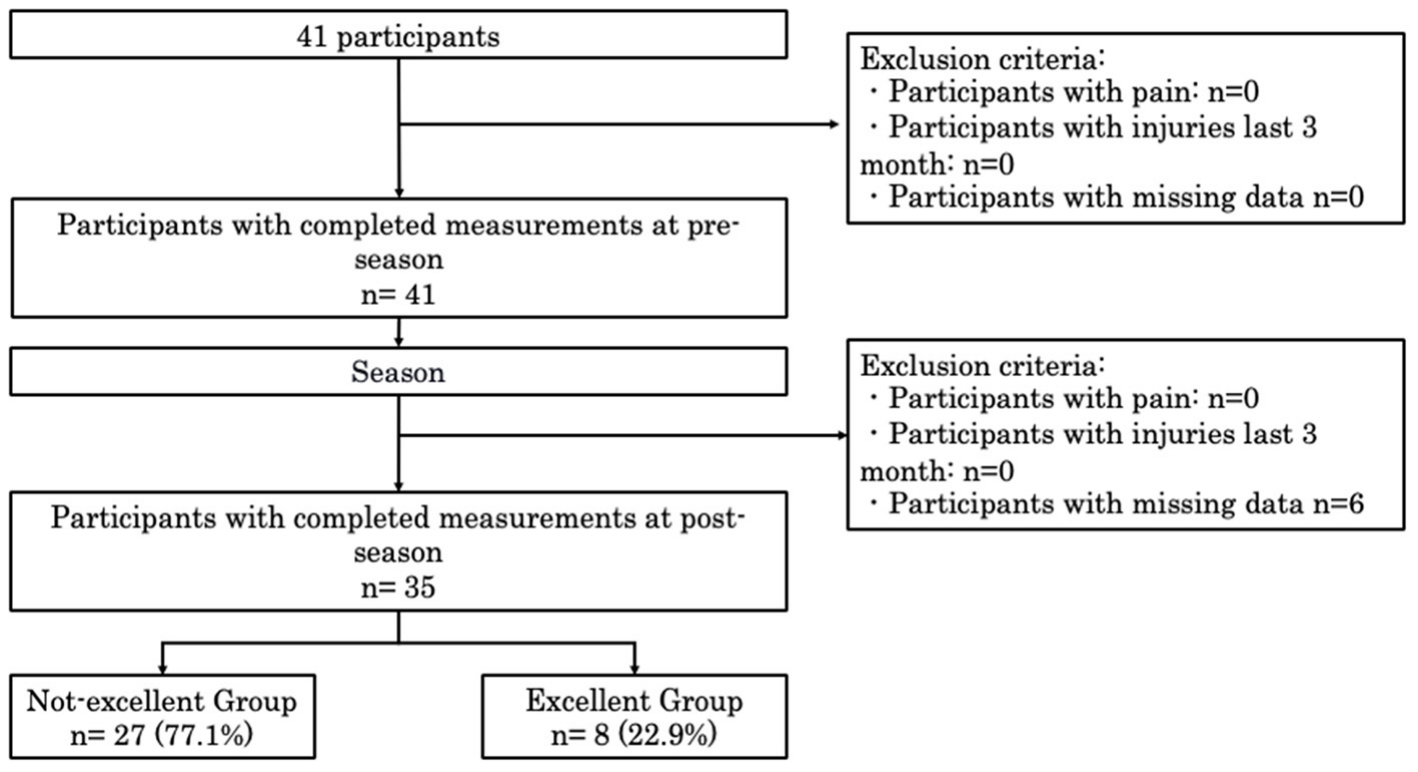
Inclusion and exclusion criteria. KA, KOJI AWARENESS.

A total of 35 runners were included in the analysis (Figure 1). Of these, eight participants (22.9%) were classified into the Excellent Awareness Group, and 27 (77.1%) into the Not-Excellent Awareness Group based on reported awareness.

**Figure 2:**
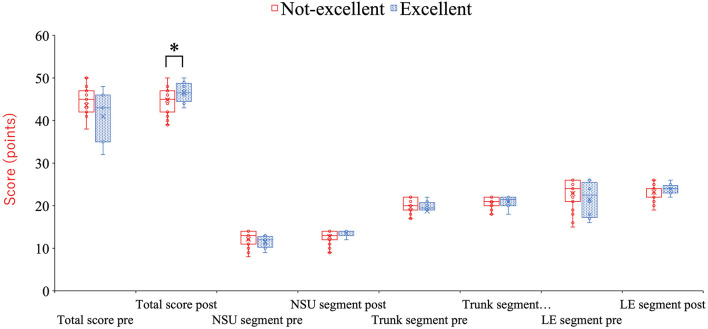
Differences in scores between groups in the total score and each segment score. ^*^p = 0.009 (compared with the non-excellent group). KA, KOJI AWARENESS; NSU, neck-scapula-upper extremity-complex; LE, lower extremity.

**Figure 3:**
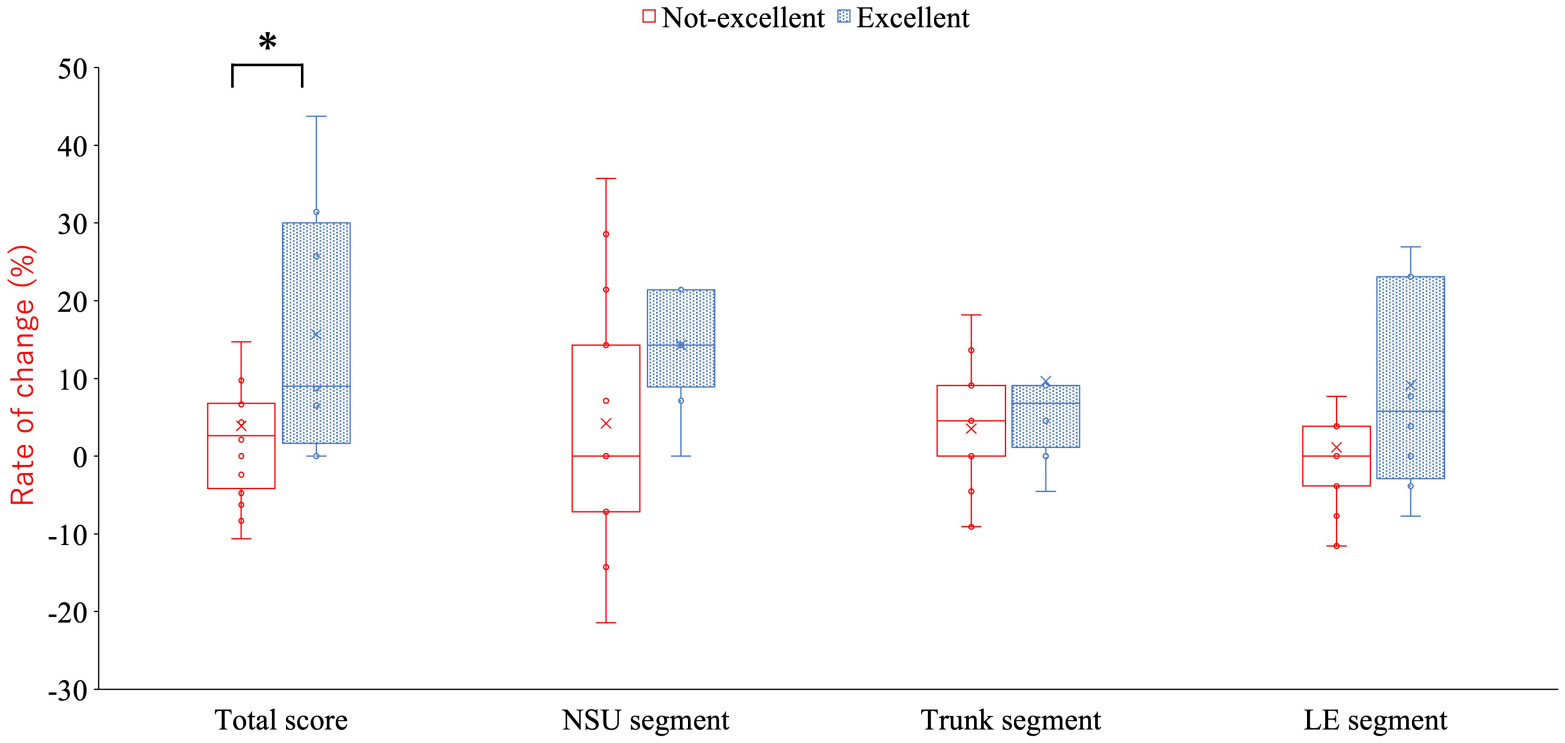
Differences between groups in seasonal change in the total score and each segment score *p = 0.009 (compared with the non-excellent group). KA, KOJI AWARENESS; NSU, neck-scapula-upper extremity-complex; LE, lower extremity.

**Figure 4:**
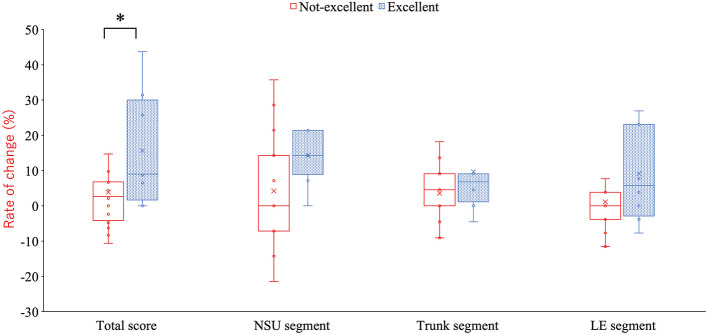
Differences between groups in the seasonal rate of change in the total score and each segment score. *p = 0.013 (compared with the non-excellent group). KA, KOJI AWARENESS; NSU, neck-scapula-upper extremity-complex; LE, lower extremity.

Baseline demographic characteristics did not differ significantly between groups (Table 2). Post-season KA total scores were significantly higher in the Excellent Awareness Group compared with the Not-Excellent Awareness Group (47.0[2.8], 46.6 ± 2.2 vs. 45.0 [4.0], 44.9 ± 3.0, *p* = 0.009) (Table 3 and Figure 2). Additionally, both the absolute and relative improvements in KA scores from pre-season to post-season were greater in the Excellent Awareness Group (absolute change: 4.0 [7.3], 5.6 ± 4.8 vs. 1.0 [4.5], 1.3 ± 3.7, p = 0.009; relative change: 9.0 [22.3], 5.6 ± 4.8 vs. 3.0 [10.0], 3.9 ± 10.6, p = 0.013) (Table 3 and Figures 3, 4).

**Table 2:** Demographic characteristics of participants.

**Status**	**Not-excellent group**	**Excellent group**	**All**	***p*-value, ES**
Male, *n*	27	8	35	–
Age, years	20.0 (3.0)	20.0 (1.3)	20.0 (2.0)	0.475, −0.06
	20.0 ± 1.2	20.0 ± 0.8	20.0 ± 1.2	
Height, cm	170.0 (8.0)	174.0 (5.3)	171.0 (6.0)	0.269, 0.53
	171.0 ± 6.1	174.0 ± 3.7	172.0 ± 5.7	
Weight, kg	55.0 (6.0)	57.0 (5.5)	56.0 (6.0)	0.294, 0.46
	56.0 ± 4.6	58.0 ± 3.4	56.0 ± 4.4	
BMI, kg/m^2^	19.0 (0.8)	19.0 (0.8)	19.0 (0.9)	0.676, 0.00
	19.0 ± 0.6	19.0 ± 1.1	19.0 ± 0.8	

Data are presented as median (interquartile range) in the upper column and mean ± standard deviation in the lower column.

BMI, body mass index; ES, effect size.

**Table 3:** Differences between groups in scores and KA change rate.

**Parameter**	**Not-excellent group**				**Excellent group**				***P*** **-value, ES**			
	**Pre**	**Post**	**Amount of change**	**Rate of change**	**Pre**	**Post**	**Amount of change**	**Rate of change**	**Pre**	**Post**	**Amount of change**	**Rate of change**
Total	45.0 (5.0), 43.6 ± 5.0	45.0 (4.0), 44.9 ± 3.0,	1.0 (4.5), 1.3 ± 3.7	3.0 (10.0), 3.9 ±1 0.6	43.0(11.0), 41.0 ± 5.7	47.0 (2.8)^*^, 46.6 ± 2.2	4.0 (7.3)^*^, 5.6 ± 4.8	9.0 (22.3)^*^, 15.7 ± 15.0	0.408, 0.50	0.009, 0.60	0.009, −0.43	0.013, −0.41
NSU segment	13.0 (2.), 12.1 ± 1.8	13.0 (2.0), 12.7 ± 1.5	0.0 (2.5), 0.6 ± 1.9	0.0 (17.9), 4.2 ± 13.8,	12.0 (1.5), 11.5 ± 1.3	14.0 (1.0), 13.5 ± 0.7	2.0 (1.3), 2.0 ± 1.0,	14.0 (1.0), 14.3 ± 7.1,	0.332, −0.22	0.304, −0.20	0.062, 0.8	0.062, 0.8
Trunk segment	20.0 (2.0), 19.7 ± 2.4	21.0 (2.0), 20.4± 1.5	1.0 (2.0), 0.8 ± 1.8	5.0 (9.1), 3.5 ± 8.1	20.0 (1.3), 18.8 ± 3.5	22.0 (2.0), 20.9 ± 1.4	2.0 (1.3), 2.1 ± 3.1	7.0 (5.7), 9.7 ± 14.3	0.576, −0.10	0.451, −0.14	0.286, −0.19	0.286, −0.19
LE segment	24.0 (4.5), 22.9 ± 3.2	24.0 (2.0), 23.2± 2.1	0.0 (2.0), 0.3 ± 2.8	0.0 (7.7), 1.1 ± 10.8	23.0 (6.8), 21.5 ± 3.8	24.0 (1.3), 23.9 ± 1.2	2.0 (6.3), 2.4 ± 3.3	6.0 (24.0), 9.1 ± 12.6	0.451, −0.13	0.603, −0.10	0.166, −0.24	0.166, −0.24

Data are presented as median (interquartile range) in the upper row and mean ± standard deviation in the lower row.

^*^p < 0.05 (compared with the non-excellent group).

KA, KOJI AWARENESS; ES, effect size; NSU, neck-scapula-upper extremity-complex; LE, lower extremity.

## 4 Discussion

### 4.1 Principal findings

This study examined the relationship between bodily awareness, functional improvement, and RRI incidence in elite long-distance runners using the KA test. Athletes with high awareness demonstrated significantly greater post-season KA scores and larger improvements from pre-season, both in absolute and relative terms. Although the between-group difference in RRI incidence was not statistically significant, the awareness group experienced no injuries during the season, compared with a 14.8% incidence in the other group. These findings partially support the hypothesis that awareness contributes to functional adaptation and may provide resilience against injury.

### 4.2 Interpretation and theoretical context

Only 22.9% of participants were classified into the Excellent Awareness Group, indicating substantial intra-team variability in bodily awareness. This is consistent with previous report that awareness differs not only across sports or experience levels but also among individuals in similar training environments (Mehling et al., 2012). Athletes with greater awareness may possess enhanced interoceptive sensitivity—the ability to perceive internal bodily states—which can promote adaptive training responses, more effective fatigue management, and lower injury risk (Murofushi et al., 2024a; Herbert and Pollatos, 2012).

Inter-individual variability in awareness may be explained by factors such as athletic experience and personality traits. Experienced athletes tend to exhibit higher interoceptive sensitivity due to long-term exposure to bodily feedback (Mehling et al., 2009). Moreover, personality traits such as conscientiousness and extraversion have been reported to promote self-monitoring and training adherence, contributing to the development of awareness (Yang et al., 2024).

Strategies to enhance awareness include proprioceptive training and mindfulness-based interventions. These approaches have been shown to improve attentional control, self-awareness, posture regulation, and balance, thereby strengthening bodily awareness and enhancing athletic performance (Yilmaz et al., 2024; Si et al., 2024). The findings of this study suggest that combining self-screening tools with corrective exercises may help increase athletes' awareness, ultimately improving functional outcomes and reducing injury risk.

These results reinforce the broader theoretical perspective that bodily awareness is not an accessory to performance but a foundation of embodied motor control. Merleau-Ponty emphasized that the body is a lived structure through which perception and action occur (Merleau-Ponty, 1962). Similarly, Heidegger's concept of being-in-the-world and Zen or Budo principles such as mushin (no-mind) and zanshin (remaining mind) describe the integration of perception, intention, and action (Heidegger, 1962). Within this framework, the KA test may function not only as a screening tool but also as a catalyst for embodied self-understanding that supports both functional improvement and injury resilience.

### 4.3 Practical implications

Although the same corrective exercise program was applied to all athletes, only those with higher awareness demonstrated notable improvements. This pattern suggests a potential role of awareness in mediating the effectiveness of exercise interventions. The KA test, therefore, may serve a dual purpose: enabling objective functional screening while prompting athletes to reflect on their physical condition. Such self-perception could encourage more engaged conditioning, greater adherence, and improved outcomes. In practice, coaches and clinicians may benefit from incorporating awareness-based tools like the KA test to tailor interventions according to athletes' levels of self-awareness, thereby enhancing intervention efficiency and potentially reducing injury risk in high-load training settings. On the other hand, the relationship between a potential role of awareness and the effectiveness of corrective exercise was not directly tested, therefore future research should include formal mediation analyses to clarify whether awareness truly mediates intervention effects.

Over the 6-month observation period, four athletes sustained RRIs, resulting in an overall incidence rate of 11.4% (Table 4). RRI incidence rates were 0% in the Excellent Awareness Group and 14.8% in the Not-Excellent Awareness Group. None of the injuries required surgical intervention. Although the number of RRIs was higher in the Not-Excellent Awareness Group (risk ratio: 2.9, 95%CI: 0.17–48.5), the between-group difference was not statistically significant (*p* = 0.553). The specific injury types and training days lost are summarized in Table 5.

**Table 4:** Rate of injury in each group.

**Athletes' condition**	**Not-excellent group, *N* = 28**	**Excellent group, *N* = 8**	**All, *N* = 35**	***P*-value, ES**	**Risk ratio (95%CI)**
Injury, *N* (%)	4 (11.4)	0 (0.0)	4 (11.4)	0.553	2.9 (0.17–48.5)
Non-injury, *N* (%)	23 (65.7)	8 (22.9)	31 (88.6)		
All, *N* (%)	27 (77.1)	8 (22.9)	35 (100.0)		

ES, effect size.

95%CI, confidence interval.

**Table 5:** Types of running-related injuries and competition and training time lost.

**Type of running injury**	**Number**	**Competition and training time lost, days^a^**
Medial tibial stress syndrome	2	48.0 (4.5)
Iliotibial band friction syndrome	1	37.0
Fibula stress fracture	1	27.0
All^a^	4	40.0 (10.8)

^a^Dates are presented as mean ± standard deviation.

### 4.4 Methodological considerations and limitations

Several limitations in this study must be acknowledged. First, the study involved a single collegiate team with a relatively small sample size, raising the possibility of selection bias and limiting the generalizability of the findings to other populations or competitive levels. However, because this team won the race two years in a row, the results of this study can be a clue for other teams to improve their performance. Second, group classification relied on self-reported awareness using a qualitative scale and did not include objective interoceptive measures such as heartbeat detection or heart rate variability. Third, other influential factors—including training load, sleep quality, and nutrition—were not assessed (Lee et al., 2017). Fourth, the absence of objective interoceptive measures limits the robustness of bodily awareness assessment. Fifth, self-reported awareness may be subject to social desirability bias, potentially inflating perceived awareness levels. Sixth, the lack of a control group without corrective exercises restricts causal inference regarding the effectiveness of the intervention. Finally, injuries were defined as those resulting in >3 weeks of missed training, which excluded mild and moderate injuries. If these injuries had been included, the overall incidence and interpretation of injury risk might have differed.

Future studies should include multi-institutional cohorts and employ multimodal assessments incorporating physiological measures such as heart rate variability, dietary intake, and recovery status. Additionally, incorporating a pre-season awareness measure and validating the awareness construct with established tools such as the Multidimensional Assessment of Interoceptive Awareness (MAIA) would strengthen methodological rigor (Mehling et al., 2012, 2009). Broadening the scope from a musculoskeletal emphasis to a holistic perspective of the athlete's body may enhance the preventive and functional value of awareness-based interventions.

## 5 Conclusion

Long-distance runners with higher bodily awareness, as assessed by the KA test, showed significantly greater functional improvements during the season. Although group differences in injury incidence did not reach statistical significance, no injuries occurred in the awareness group. These findings suggest that conscious awareness of physical function may enhance the impact of corrective exercise interventions and support physical maintenance throughout competitive periods.

## Data Availability

The raw data supporting the conclusions of this article will be made available by the authors, without undue reservation.
