# Supplementary material for: Bodily awareness predicts functional improvement and injury risk in elite long-distance runners: a prospective study
Source: Front Psychol. 2025 Dec 17;16:1718718. doi: 10.3389/fpsyg.2025.1718718 (PMC12753356; doi:10.3389/fpsyg.2025.1718718)
Supplement: Supplementary file 1 [file Supplementary_file_1.pdf]

KOJI AWARENESS™ corrective exercises

1. Archer’s rotation

For neck mobility

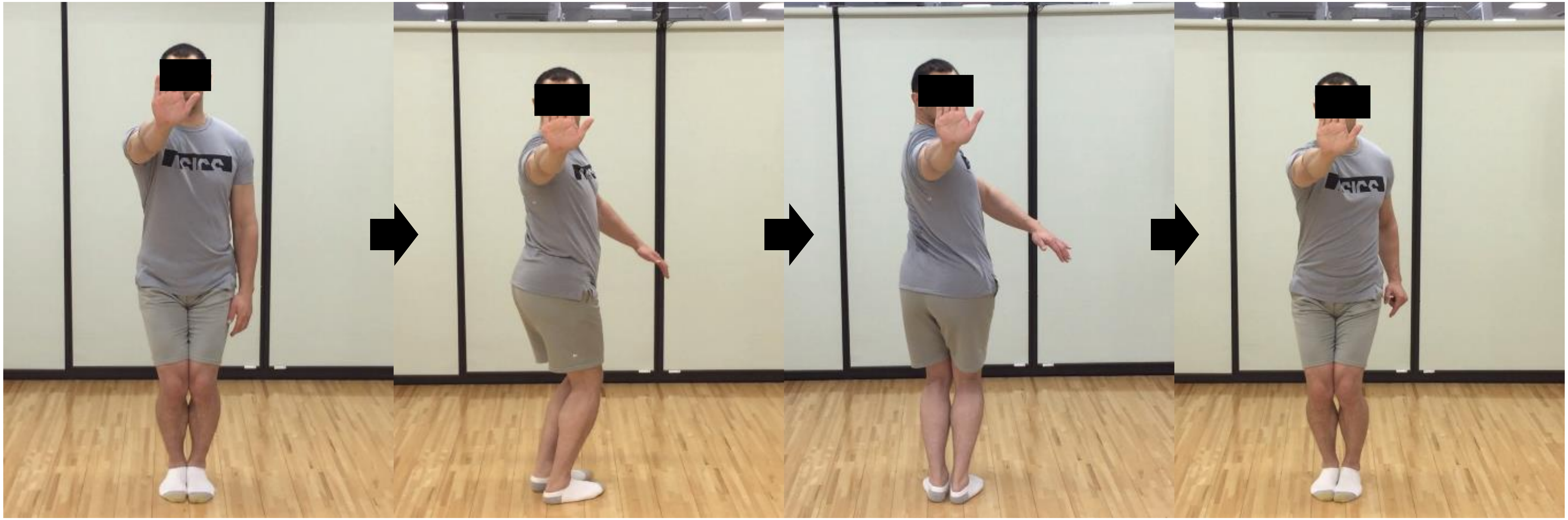

1. Bend your knees and keep your upper body straight and try to keep facing the back of your hand while you rotate.
2. Take tiny footsteps to one side until you cannot turn any further.
3. Take tiny footsteps again to come back to the start position.

*MAKE SURE to keep facing the back of your hand stays in place. If you can stay lower, it would also be a good exercise for the hip and thighs.

2. Python Squeeze
For neck mobility

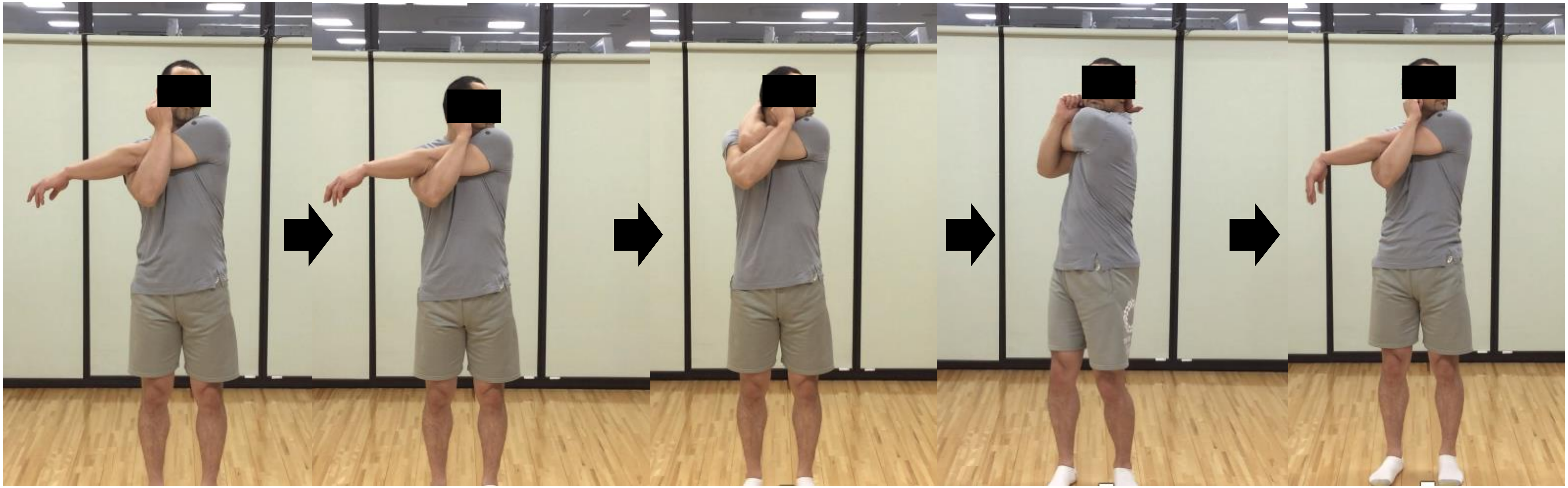

1. Stand still, arm straight and bring with other arm across and curl towards to your self.
2. Head rotate to the direction of arm extended.
3. Bend arm and reach behind of your head with back of your hand (or reach ears if you are flexible enough).
4. Twist whole upper body towards behind head. Hold for few second, unwind and relax whole body.

*MAKE SURE to move slow as you can and feel the stretch. Breath correctly while doing exercise.

3. Wall reverse push

For shoulder mobility

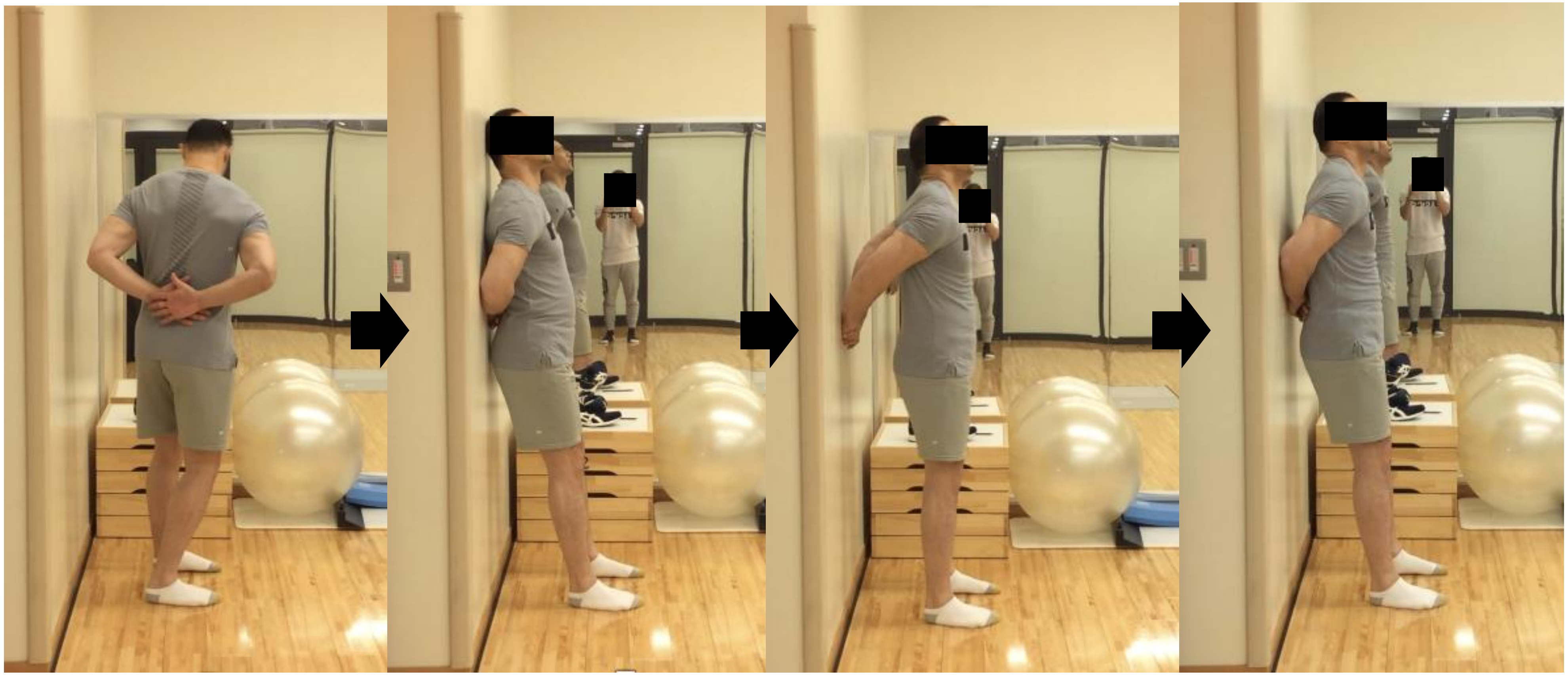

1. One foot away from the wall and face away. Hands together and put your back of the hands on your back, lean back against the wall.
2. Slowly push yourself away from the wall and come back towards the wall.

*MAKE SURE to keep your body in a straight line during exercise. The targeting shoulder should be on the same side as the hand in contact with the wall.

4. Wall angel slider

For scapula mobility

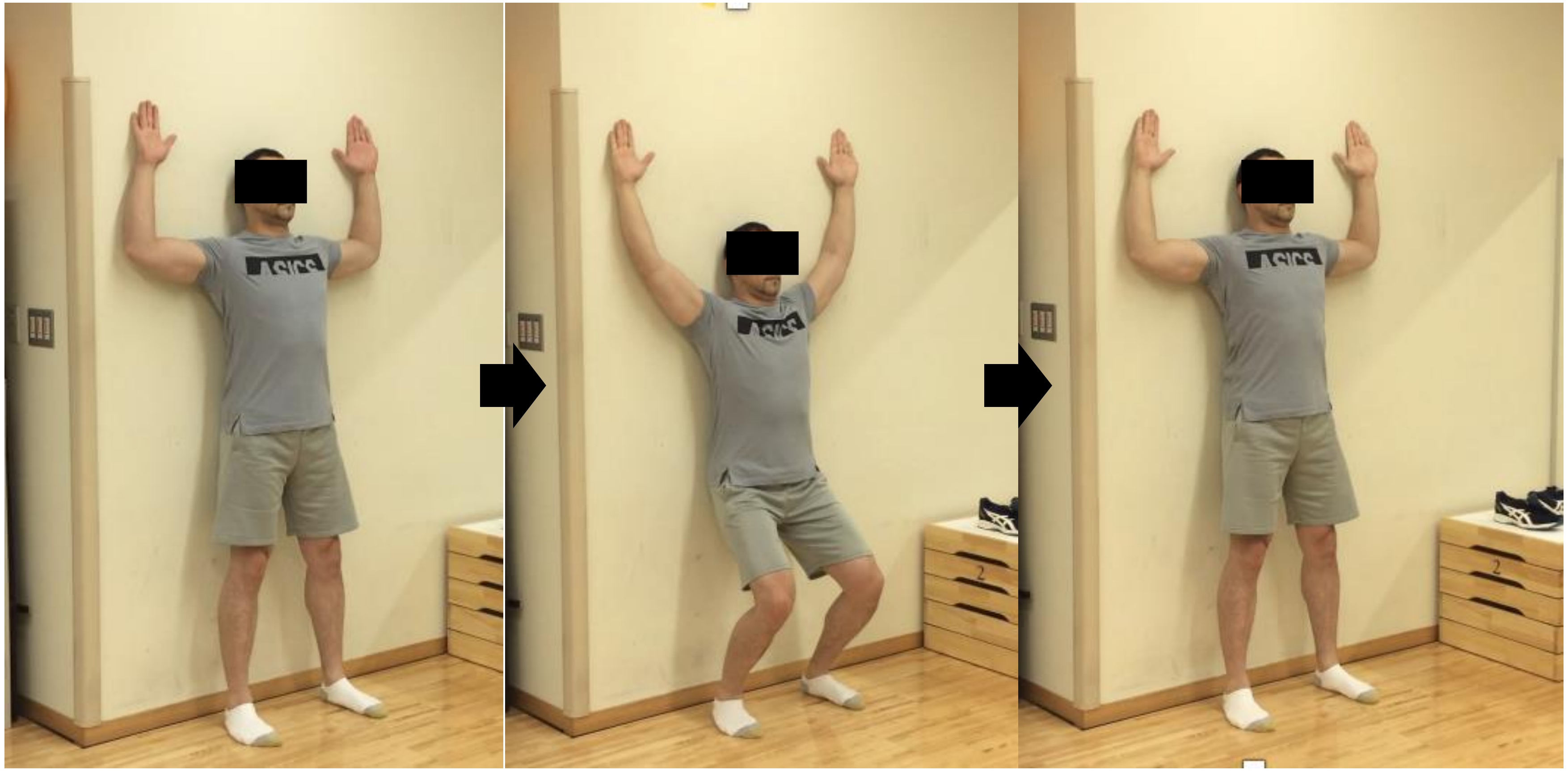

1. One knuckle away from the wall and face away. Set shoulder parallel, elbow 90 degrees to the ground, lean back against the wall.
2. Try not to make space between your back and the wall.
3. Bend your knees and slowly slide down against the wall.
4. Stop when you cannot keep your elbow and back from the wall and come back slowly to the beginning of the position.

*MAKE SURE not to move your wrist while sliding.

5. Flamenco thoracic exercise

For thoracic spine mobility

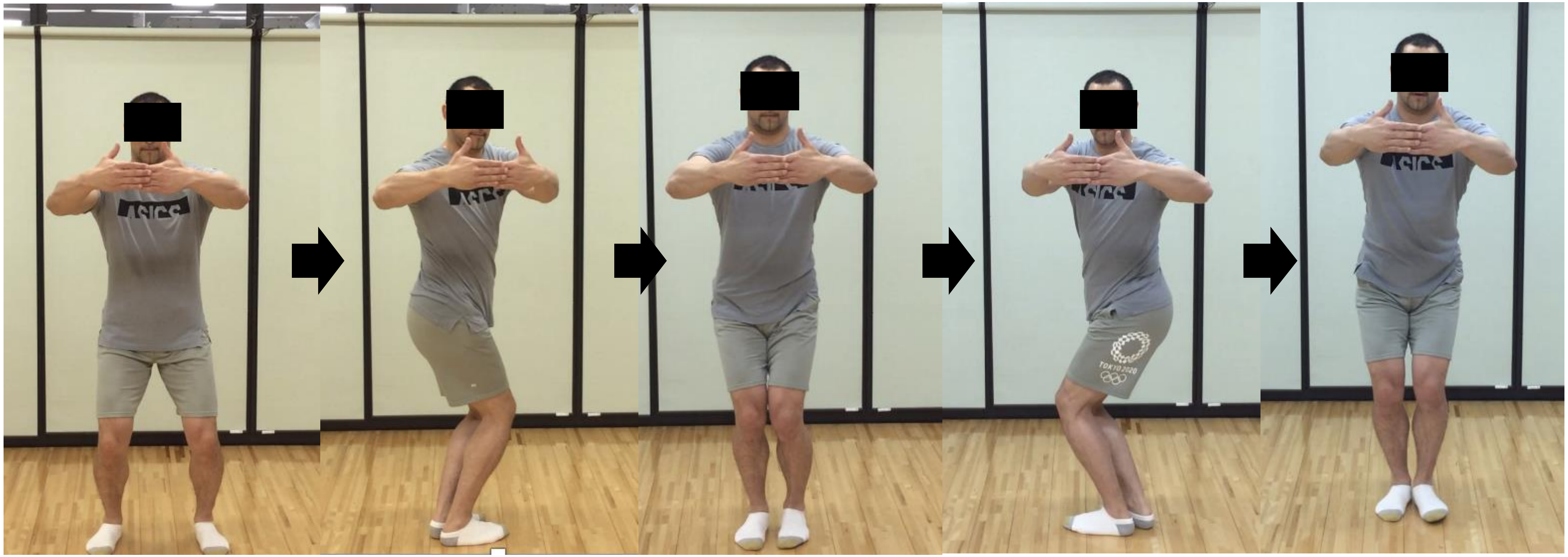

1. Bend your knees and keep your upper body straight and still while making a circle with your arms.
2. Take tiny footsteps to one side until you cannot turn any further.
3. Take tiny footsteps again to come back to the start position.

*MAKE SURE to maintain your upper body and circled arms stay in place. If you can stay lower, it would be a good exercise for the hip and thighs.

6. Weight shift wall push

For upper extremity stability & strength

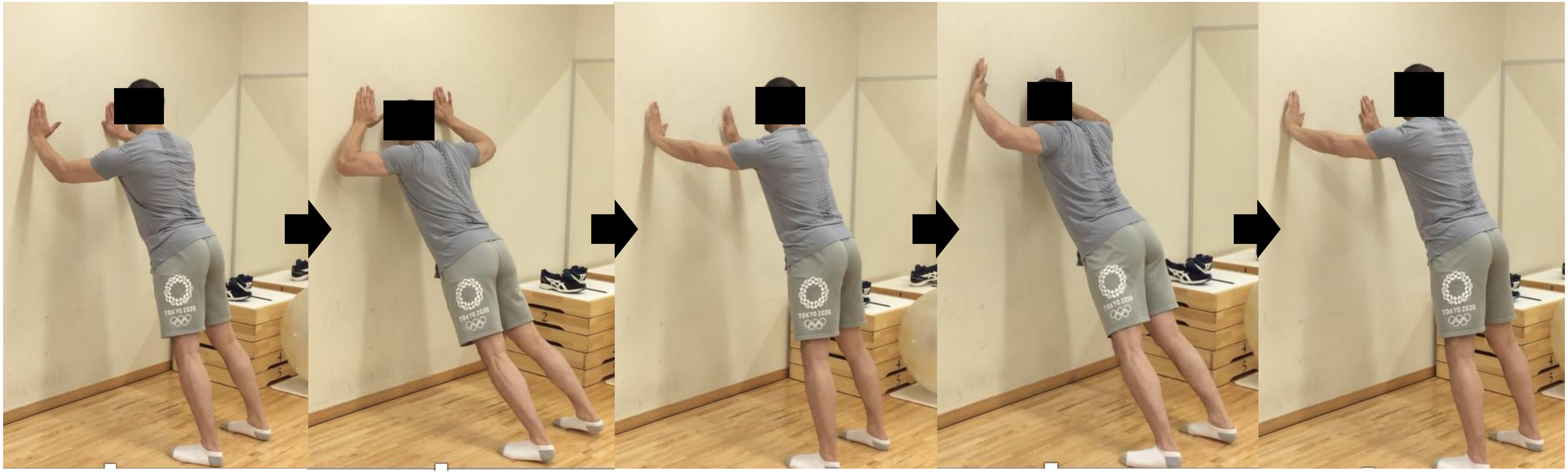

1. Stand four foot away from the wall and create a front hand-plank position on the wall. Hands were positioned shoulder-width at the level of eyes.
2. Move your body closer to the wall.
3. Push up against the wall, but switch pressure on your palm, ulnar, and radial side every time (for example, right hand: ulnar side, left hand: radial side. Change the sides every time).

7. Side sitting to lift

For hip mobility

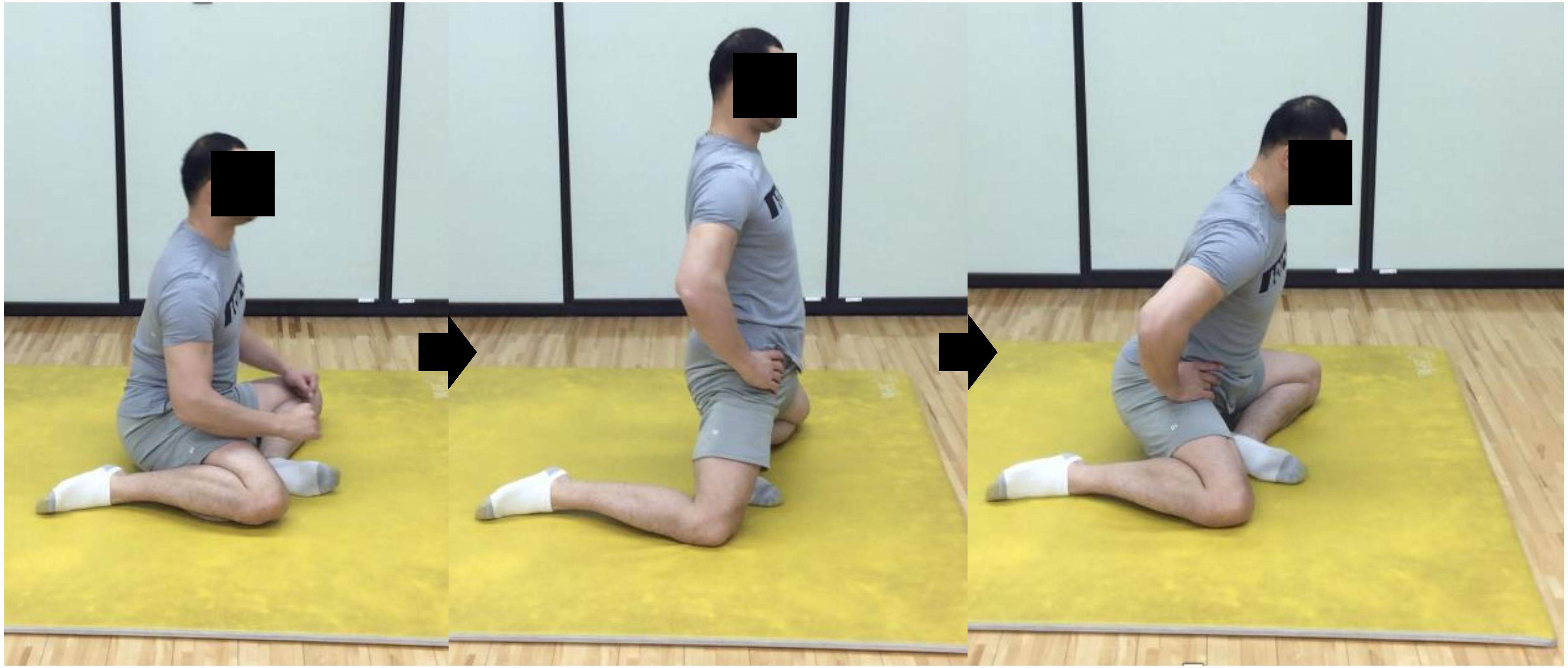

1. Sitting on the floor, in a side sitting position, leg folded back to one side.
2. Start to lift the pelvis stay tall on the knee. Squeeze your glutes.

*MAKE SURE to open up and feel the stretch of the hip flexor every time.

8. Weight shift squat

For hip mobility

For lower extremity strength

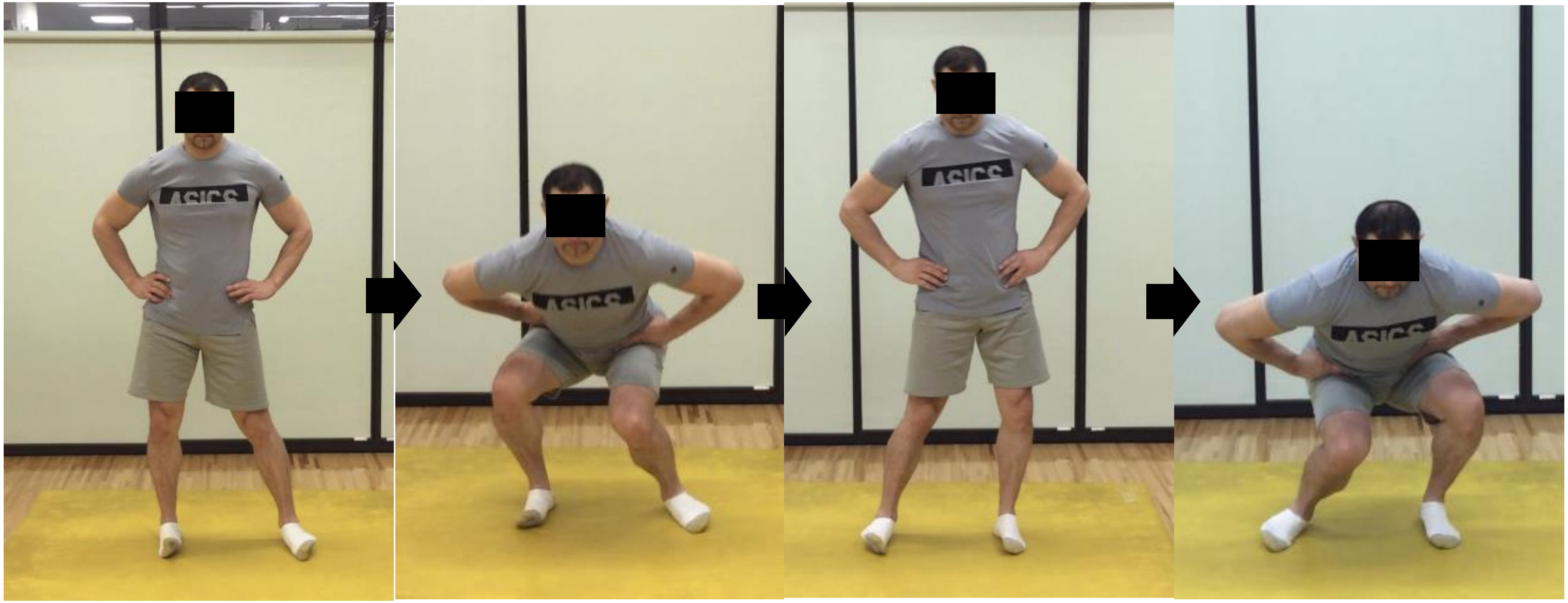

1. Stand with your feet shoulder-width apart.
2. Start squatting with a weight shift each time, put weight on the outside and inside of the each foot (for example, putting weight on the outside of the right foot and inside of the left foot).

*MAKE SURE to keep the pelvis parallel to the ground, straight and try not to rotate your lower body while sitting down.

9. Straight leg lowering Exercise

For hip and spine mobility (Bening forward)

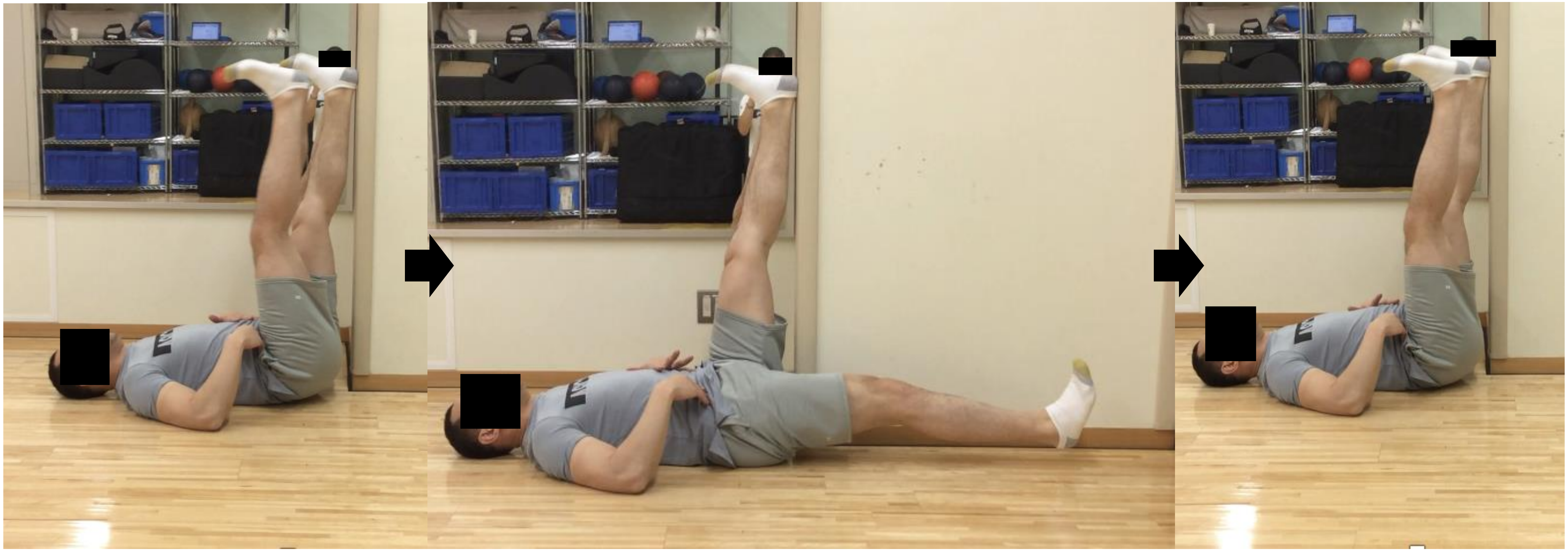

1. Lie on the floor and put one leg on the wall.
2. Lower the other leg closer to the floor while maintain knee extension and ankle dorsiflexion.
3. Slowly back to the start position.

*MAKE SURE to move your leg slowly, breathe slowly to feel the stretch of your hamstrings.

10. Single-leg squat with an ankle hold

For hip and spine mobility (Bending backward)

For upper and lower extremity, mobility & stability

For power extremity strength

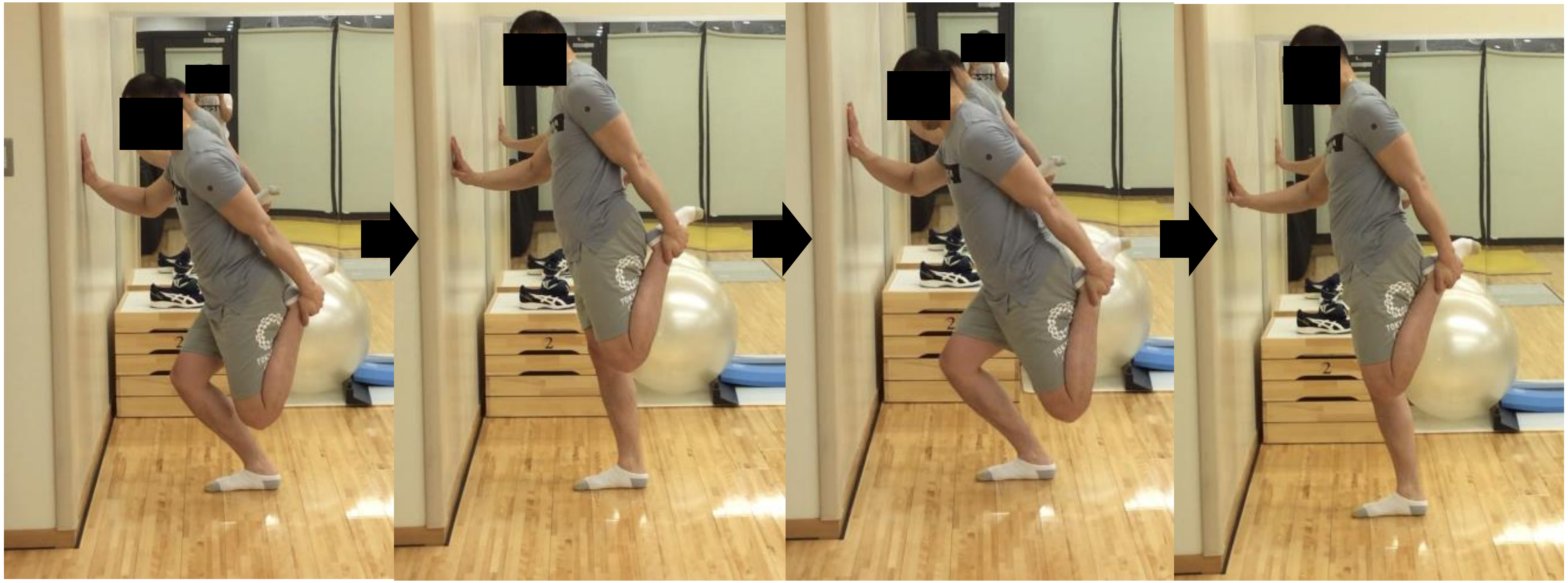

1. Hold the ankle and keep the heel on the buttock.
2. Flex the knee and hip of the supported leg and lower the body while holding the other leg.
3. Extend the knee and hip of the supported leg and back to start position.

*MAKE SURE to squeeze your buttock (isometric contraction) while extending the leg.

11. Straight leg lowering 45

For mid-section stability strength

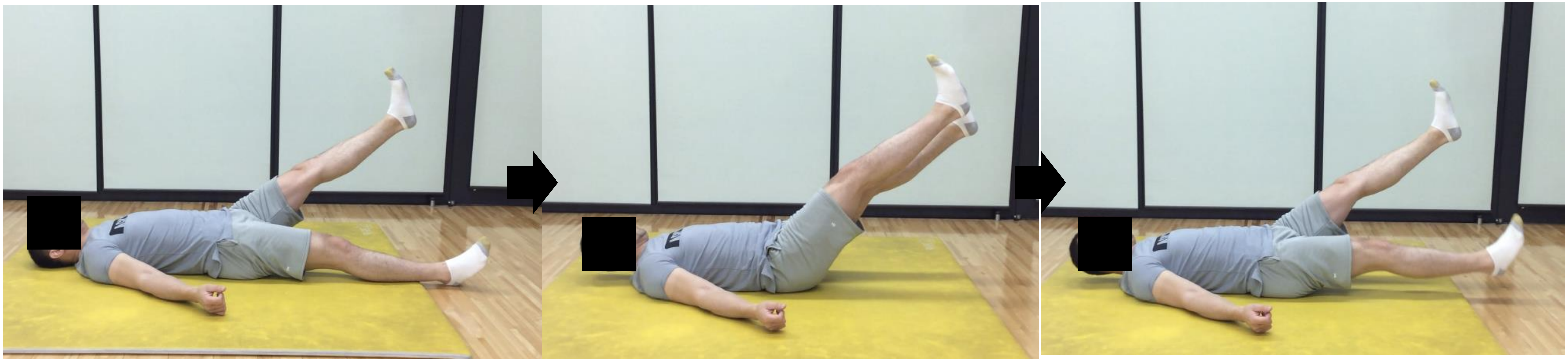

1. Lie on the floor and lift one leg with knee extension about 45 degrees.
2. Lift the other leg to same height of the lifted leg and lower slowly.
3. Repeat the movements.

*MAKE SURE to move slowly and moving leg of the heel should not touch on the floor.

12. Koji wall push
For ankle mobility

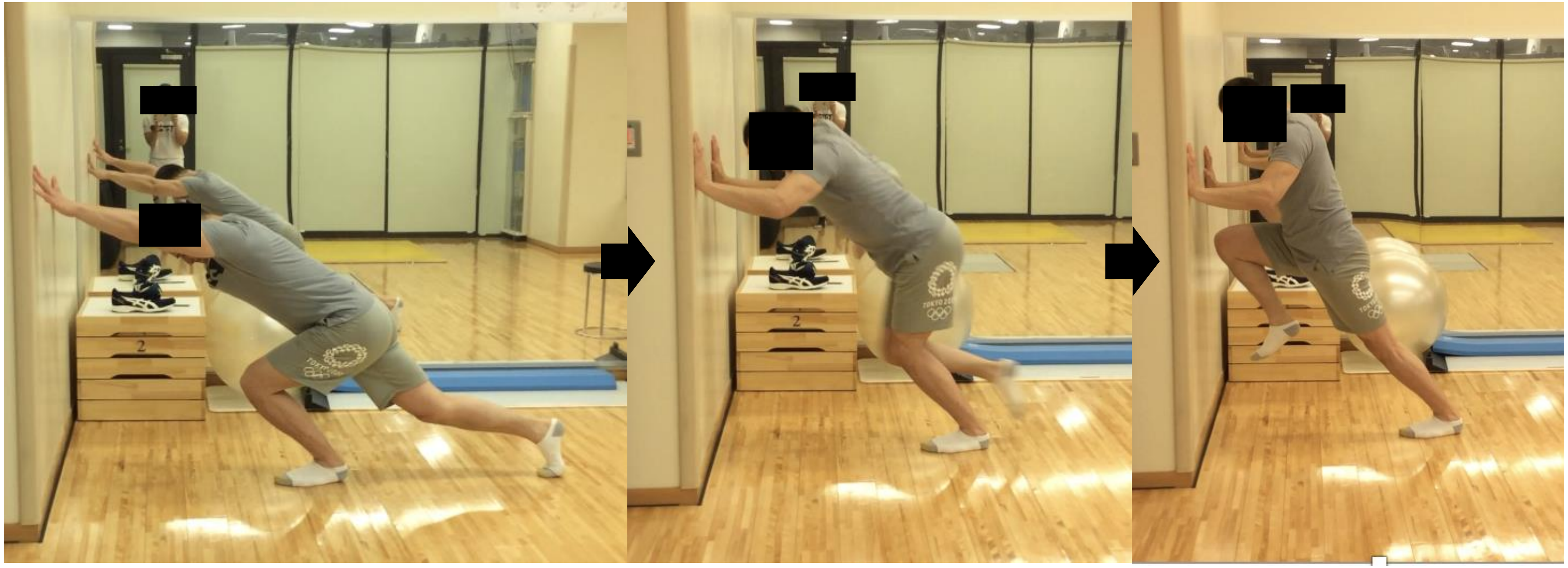

1. Stand 3-4 steps away from the wall and put both hands on the wall at the face level.
2. Draw one foot backward and bend the other leg’s knee as much as possible with sole on the floor.
3. Lift the leg at backward until the thigh is parallel to the floor.

*MAKE SURE to stay low when you start, push the wall, and put your heel down on the ground
